# The *Arabidopsis* NIMIN proteins affect NPR1 differentially

**DOI:** 10.3389/fpls.2013.00088

**Published:** 2013-04-12

**Authors:** Meike Hermann, Felix Maier, Ashir Masroor, Sofia Hirth, Artur J. P. Pfitzner, Ursula M. Pfitzner

**Affiliations:** FG Allgemeine Virologie, Institut für Genetik, Universität HohenheimStuttgart, Germany

**Keywords:** NIM1-INTERACTING (NIMIN) proteins, *NON-EXPRESSOR OF PATHOGENESIS-RELATED GENES1* (*NPR1*), *PATHOGENESIS-RELATED GENE1* (*PR-1*), plant defense gene activation, protein–protein interaction, salicylic acid (SA), systemic acquired resistance (SAR)

## Abstract

NON-EXPRESSOR OF PATHOGENESIS-RELATED GENES1 (NPR1) is the central regulator of the pathogen defense reaction systemic acquired resistance (SAR). NPR1 acts by sensing the SAR signal molecule salicylic acid (SA) to induce expression of *PATHOGENESIS-RELATED* (*PR*) genes. Mechanistically, NPR1 is the core of a transcription complex interacting with TGA transcription factors and NIM1-INTERACTING (NIMIN) proteins. *Arabidopsis* NIMIN1 has been shown to suppress NPR1 activity in transgenic plants. The *Arabidopsis* *NIMIN* family comprises four structurally related, yet distinct members. Here, we show that *NIMIN1*, *NIMIN2*, and *NIMIN3* are expressed differentially, and that the encoded proteins affect expression of the SAR marker *PR-1* differentially. *NIMIN3* is expressed constitutively at a low level, but *NIMIN2* and *NIMIN1* are both responsive to SA. While *NIMIN2* is an immediate early SA-induced and *NPR1*-independent gene, *NIMIN1* is activated after *NIMIN2*, but clearly before *PR-1*. Notably, *NIMIN1*, like *PR-1*, depends on *NPR1*. In a transient assay system, NIMIN3 suppresses SA-induced *PR-1* expression, albeit to a lesser extent than NIMIN1, whereas NIMIN2 does not negatively affect *PR-1* gene activation. Furthermore, although binding to the same domain in the C-terminus, NIMIN1 and NIMIN2 interact differentially with NPR1, thus providing a molecular basis for their opposing effects on NPR1. Together, our data suggest that the *Arabidopsis* NIMIN proteins are regulators of the SAR response. We propose that NIMINs act in a strictly consecutive and SA-regulated manner on the SA sensor protein NPR1, enabling NPR1 to monitor progressing threat by pathogens and to promote appropriate defense gene activation at distinct stages of SAR. In this scenario, the defense gene *PR-1* is repressed at the onset of SAR by SA-induced, yet instable NIMIN1.

## INTRODUCTION

Plants have evolved different layers of defense to recognize and combat invading microbes (Jones and Dangl, 2006). The immune response systemic acquired resistance (SAR) is launched after primary infection and activation of effector-triggered immunity (ETI) accompanied by formation of necrosis at the sites of pathogen invasion. SAR becomes effective in non-infected plant tissue far away from the pathogen penetration sites (Ross, 1961). The response fends off secondary infections by diverse types of biotrophic pathogens and is long-lasting. The local signal to induce SAR in non-infected leaves is salicylic acid (SA;Vernooij et al., 1994). Levels of free and conjugated SA rise not only in infected necrotic tissue, but also systemically in non-infected leaves (Malamy et al., 1990;Métraux et al., 1990). This increase in SA concentration is paralleled by local and systemic induction of various *PATHOGENESIS-RELATED* (*PR*) genes (van Loon and van Kammen, 1970;Ward et al., 1991;van Loon et al., 2006). Some *PR* genes, e.g., *PR-1*, can be induced solely by exogenous application of SA or its functional analogs 2,6-dichloroisonicotinic acid (INA) and benzo(1,2,3)thiadiazole-7-carbothioic acid *S*-methyl ester (BTH;White, 1979;Vernooij et al., 1995;Friedrich et al., 1996;Lawton et al., 1996). Furthermore, it has been shown that SA-treated tobacco and *Arabidopsis* plants expressing *PR-1* genes display SAR (White, 1979;Uknes et al., 1992, 1993). Thus, accumulation of *PR-1* transcripts and PR-1 proteins either in non-infected parts of plants exhibiting necrosis or in response to exogenous application of SA serves as marker for the SAR resistance reaction.

The central regulator of SAR is *NON-EXPRESSOR OF PATHOGENESIS-RELATED GENES1* (*NPR1*). The gene was identified from *Arabidopsis* mutants compromised in chemical induction of *PR* genes and in resistance to fungal infection (Cao et al., 1997;Ryals et al., 1997;Shah et al., 1997). Overexpression experiments strongly suggest that NPR1 is active only after SA induction (Cao et al., 1998;Friedrich et al., 2001). The *Arabidopsis* NPR1 family encompasses six members, NPR1 to NPR6, and recent evidence indicates that SA signals directly through some members. However, the mechanism of how SA acts on NPR1 family proteins is controversial. First, it has been demonstrated that NPR1 from *Arabidopsis* (At) and two NPR1 family members from tobacco (Nt) alter some of their biochemical capabilities in response to the SA signal molecule in a heterologous yeast system in absence of any other plant protein (Maier et al., 2011). For example, Nt NPR1 gains transcription activity, when SA is added to yeast growth medium. The data indicate that NPR1 family proteins are able to sense SA, and that they undergo an alteration upon perception of SA. Consequently, *Arabidopsis* NPR1 family members have been found to bind SA *in vitro* (Fu et al., 2012;Wu et al., 2012), albeit with very different affinities. While NPR4 is a high affinity receptor and NPR3 is a lower affinity receptor, SA appears to bind only very weakly to NPR1. It has been proposed that *PR-1* gene activation in the course of SAR is regulated through availability of NPR1, which, in turn, is controlled by cytoplasmic oligomer–nuclear monomer shuttling and by differential interaction of NPR1 with SA-perceiving NPR4 and NPR3 in the nucleus (Mou et al., 2003;Fu et al., 2012). In two other models, SA perception during SAR has, however, been attributed to the NPR1 protein, itself.Wu et al. (2012) have suggested that NPR1 binds SA via the transition metal copper in a complex with two cysteine residues, Cys-521 and Cys-529, and that, upon SA binding, a C-terminal transactivation domain is released from the N-terminal autoinhibitory BTB/POZ (broad complex, tramtrack, and bric à brac/pox virus and zinc finger) domain. Curiously, only *Arabidopsis* NPR1 contains two closely spaced cysteine residues in its C-terminus. In a third model, based on biochemical evidence obtained in the heterologous yeast system, two distinct domains in the C-terminus of NPR1 proteins have been implicated in sensing the SA signal (Maier et al., 2011). These domains are highly conserved in NPR1 proteins from diverse species and they are also conserved in the NPR1 paralogs NPR2, NPR3, and NPR4 from *Arabidopsis* and in tobacco NPR3 (also known as NIM1-LIKE1). One domain comprises the penta-amino acid motif LENRV (amino acids 429–433). The LENRV motif imposes SA sensitivity on NPR1 proteins from *Arabidopsis* and tobacco in yeast. The signature is altered in the non-functional *nim1-4* mutant (R432K;Ryals et al., 1997). The latter model is corroborated by genetic evidence provided through an *en masse in planta* screen for *Arabidopsis* insensitive to the functional SA analog BTH (Canet et al., 2010). In this screen, dozens of *npr1* alleles were identified, and the mutants have been found to be clustered in the same two regions identified independently by biochemical dissection of NPR1 family proteins in yeast. The *nim1-4* mutant was isolated three times. On the contrary, Cys-521 and Cys-529 were not uncovered genetically.

The SA sensor protein NPR1 interacts with two groups of proteins. TGA transcription factors connect NPR1 with SA-responsive *as-1*-like *cis*-acting elements present in the promoters of *PR-1* genes from tobacco and *Arabidopsis* (Lebel et al., 1998;Strompen et al., 1998;Zhang et al., 1999;Després et al., 2000;Zhou et al., 2000). This finding is consistent with several reports showing that NPR1 proteins from *Arabidopsis*, tobacco, and rice promote transcription activation in diverse systems (Rochon et al., 2006;Maier et al., 2011;Chern et al., 2012). The data imply that NPR1 is the core of a transcription complex on *PR* gene promoters. In addition to TGA factors, NPR1 interacts with the group of small NIM1-INTERACTING (NIMIN) proteins (Weigel et al., 2001). Like *NPR1*, *NIMIN* genes are dispersed in the whole plant kingdom (Chern et al., 2005;Zwicker et al., 2007). NIMIN proteins harbor nuclear localization signals, and thus target NPR1 in the nucleus (Weigel et al., 2001;Chern et al., 2005;Zwicker et al., 2007). However, their functional significance was not evident, when NIMINs were first identified.

*Arabidopsis* contains four *NIMIN* genes, *NIMIN1*, *NIMIN1b*, *NIMIN2*, and *NIMIN3* (Weigel et al., 2001). Of these, *NIMIN1* and *NIMIN2* have been studied in some detail. Both genes are strongly up-regulated by SA. In contrast, the two genes are not induced significantly in pathogen-infected necrotic tissue displaying ETI (Glocova et al., 2005). Hence, *NIMIN1* and *NIMIN2* seem to be specifically linked to the SA-dependent SAR response, rather than to ETI. Similarly, tobacco *NIMIN2*-type mRNAs accumulate in response to the SA signal molecule (Horvath et al., 1998;Zwicker et al., 2007). Although clearly structurally related, the *Arabidopsis* NIMIN proteins are distinct from each other. For example, they interact differentially with NPR1 (Weigel et al., 2001). NIMIN3 interacts with the At NPR1 N-terminal half, whereas NIMIN1, NIMIN1b, and NIMIN2 possess similar motifs by which they bind to the At NPR1 C-terminal third. In the C-terminus of Nt NPR1, the binding region of SA-induced NIMIN2-type proteins has been mapped from amino acids 494 to 510 (Maier et al., 2011). Notably, several *npr1* mutant alleles have been uncovered in the corresponding region of At NPR1, all of which affect responsiveness to BTH *in planta* (Canet et al., 2010). Furthermore, occurrence of the interaction domain for inducible NIMIN2-type proteins and the LENRV domain is coincident in NPR1 proteins and its paralogs from many species. Thus, these two domains appear to be intimately connected with the SA response.

The functional significance of NIMIN proteins for NPR1 activity has been addressed in overexpression experiments. Both *Arabidopsis* NIMIN1 and NEGATIVE REGULATOR OF DISEASE RESISTANCE (NRR), a NIMIN homolog from rice, are able to suppress induction of *PR* genes and to cause enhanced susceptibility to bacterial pathogens in transgenic plants (Chern et al., 2005, 2008;Weigel et al., 2005). From these data, it has been concluded that NIMIN proteins are repressors of NPR1. However, in tobacco, constitutive overexpression of Nt NIMIN2a produced only a delay in PR-1 protein accumulation, and it has been suggested that NIMIN proteins, although negatively affecting NPR1 activity, are, at bottom, positive regulators of NPR1-mediated *PR* gene induction (Zwicker et al., 2007). Apart from NIMIN1, the biological significance of other *Arabidopsis* NIMIN family members has not yet been addressed. Here, we provide evidence that the *Arabidopsis* NIMIN proteins affect NPR1 differentially at distinct stages of SAR, thus enabling the plant to strictly control defense gene activation in tissue distant from sites of pathogen entry undergoing ETI.

## RESULTS

### *NIMIN3* IS NOT RESPONSIVE TO PLANT DEFENSE SIGNALS

**FIGURE 1:**
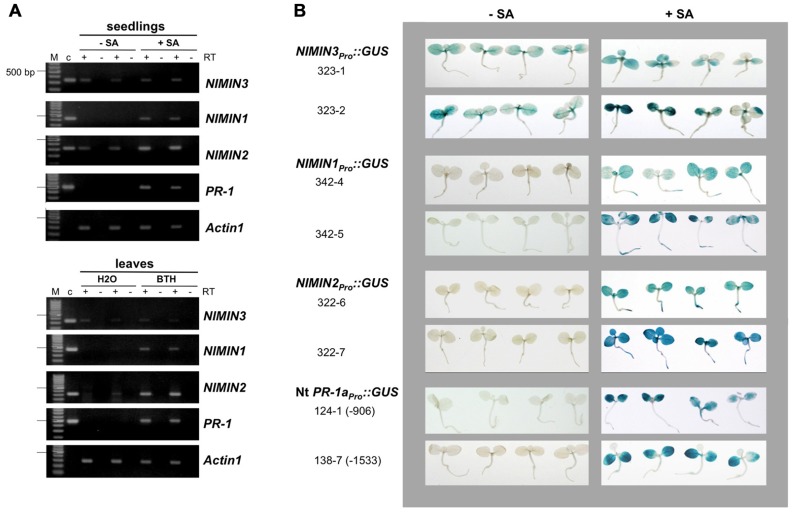
***ArabidopsisNIMIN3* is expressed constitutively**. **(A)** RT-PCR analyses of *NIMIN3* expression in *Arabidopsis* whole seedlings and leaf tissue. Expression of *NIMIN3* is compared to expression of *NIMIN1*, *NIMIN2*, and *PR-1*. RNA samples were isolated from 2-week-old whole seedlings grown either on MS medium or MS medium with addition of 0.3 mM SA and from leaves of 4-week-old plants 24 h after spraying with water or a suspension of Bion^®^ containing 0.34 mM BTH. RT-PCR analyses were performed on DNase I-treated total RNA preparations in presence or absence of reverse transcriptase (RT) with primer combinations listed in **Table 1**. In lanes c, PCR products from 1 ng of plasmid DNAs carrying the respective cDNAs were loaded. The amplification of* Actin1* mRNA serves as an internal standard for different RNA samples used in the amplification reactions. **(B)** Expression of a *NIMIN3_Pro_*::GUS reporter gene in transgenic tobacco seedlings. Expression from the *NIMIN3* promoter is compared to reporter gene expression from the *NIMIN1*, *NIMIN2*, and Nt *PR-1a* promoters. Tobacco seedlings (T1 generation) transformed with the indicated reporter genes were grown on MS medium with kanamycin or on selective medium supplemented with 0.3 mM SA. Two independent lines for each construct or, as in case of the Nt *PR-1a* promoter, two different constructs were analyzed. Seedlings were stained for GUS reporter enzyme activity when 4-weeks-old.

**FIGURE 2:**
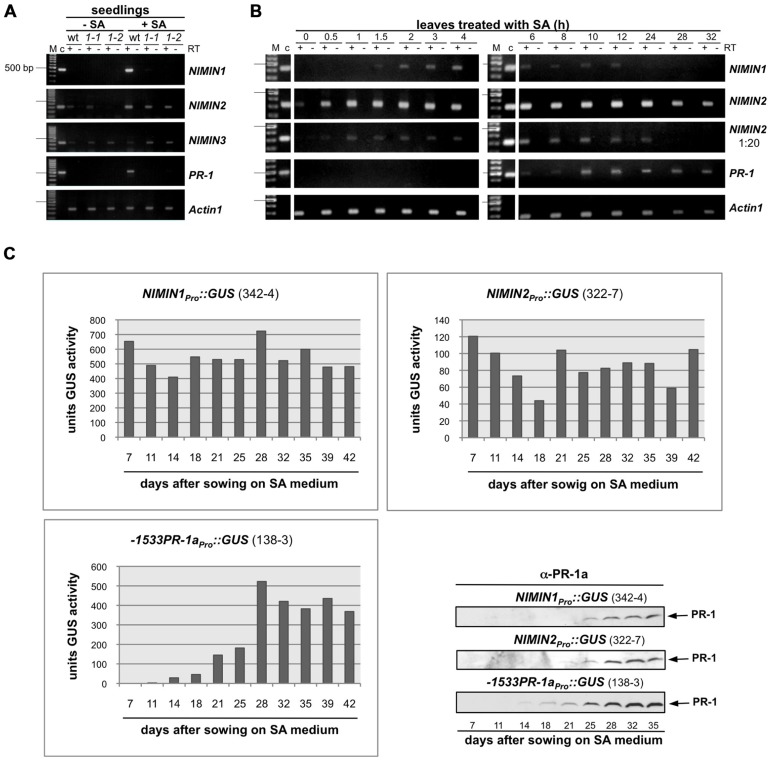
**Salicylic acid-induced *ArabidopsisNIMIN1* and *NIMIN2* are expressed differentially from each other and from *PR-1***. RNA samples were isolated from *Arabidopsis* seedlings or *Arabidopsis* leaves and analyzed as described in **Figure 1A**. Expression of *NIMIN1 *and* NIMIN2* is compared to expression of *NIMIN3* and *PR-1*.** (A) **RT-PCR analyses of RNAs from wild-type (Col-0) and *npr1-1* and *npr1-2* mutant seedlings. *1-1, npr1-1*; *1-2, npr1-2*. **(B)** RT-PCR analyses of RNAs from leaf tissue at different times after spraying plants with 1 mM SA. **(C)** Time course of SA-induced GUS reporter enzyme activities and PR-1 protein accumulation in tobacco seedlings transformed with *NIMIN1_Pro_*::GUS or *NIMIN2_Pro_*::GUS. Expression from the two *NIMIN* promoters is compared to reporter gene expression from the Nt *-1533PR-1a* promoter. For immunodetection of endogenous PR-1 proteins, equal amounts of protein were loaded in each lane of the SDS gels. Seedlings (T1 generation) were grown on selective medium with 0.3 mM SA. Similar results were obtained with independent lines of *NIMIN1_Pro_*::GUS, *NIMIN2_Pro_*::GUS and *-1533PR-1a_Pro_*::GUS.

Previously, we have shown that *NIMIN1* and *NIMIN2* are strongly induced by treatment of *Arabidopsis* plants with SA or Bion^®^, a commercial plant growth regulator containing the functional SA analog BTH, and that this induction is due to transcriptional gene activation (Weigel et al., 2001, 2005;Glocova et al., 2005). To further elucidate the functional relevance of *NIMIN* genes, we have now analyzed expression of *NIMIN3* in response to diverse signal molecules involved in plant defense reactions. Initially, transcript accumulation was monitored using reverse transcriptase-polymerase chain reaction (RT-PCR) analyses. The primers used and the sizes of fragments generated by PCR from plasmids carrying cDNAs for *NIMIN3* and various control genes are listed in **Table 1**. *NIMIN3* transcript levels were compared to expression of the *NIMIN1*, *NIMIN2*, and *PR-1* genes. Unlike *NIMIN1*, *NIMIN2*, and *PR-1*, expression of *NIMIN3* was neither induced by SA nor BTH (**Figure 1A**). Moreover, jasmonate (JA), another plant defense signal, had no effect on either of the *NIMIN* genes (data not shown). However, we were able to detect *NIMIN3* transcripts in several independent RNA preparations irrespective of whether they had been isolated from control or chemically induced plant tissue (**Figures 1A** and **2A**), suggesting that *NIMIN3* may be expressed constitutively at a low level. To address this question, we isolated 1.4 kb of the *NIMIN3* 5′-upstream region and fused it to the β -glucuronidase (*GUS*) reporter gene. The chimeric gene was transferred to the tobacco genome, and GUS enzyme activity was determined in seven independent primary transformants, all containing intact copies of the reporter gene construct (data not shown). As compared to transgenic tobacco plants carrying analogous *NIMIN1*_Pro_::GUS or *NIMIN2_Pro_*::GUS constructs (0.8 and 0 GUS units on an average, respectively;Glocova et al., 2005), untreated plants containing *NIMIN3_Pro_*::GUS exhibited constitutive GUS enzyme activity (14.7 GUS units on an average; **Figure 1B**). Reporter gene expression from the *NIMIN3_Pro_*::GUS construct in the tobacco genome was not enhanced significantly by treatment of plants with SA (0.3 and 1 mM; 17.6 GUS units on an average), BTH (0.34 mM), methyl JA (MeJA; 0.1 mM), or H_2_O_2_ (0.1 and 1 mM; data not shown). Likewise, gene expression from the *NIMIN3* promoter was not elevated by elicitation of HR or by exogenous application of the phytohormones 2,4-dichlorophenoxy acetic acid (2,4-D), gibberellic acid (GA), indole-3-acetic acid (IAA), or 1-naphthalene acetic acid (NAA; 0.01 and 0.1 mM each; data not shown). As determined by histochemical staining, *NIMIN3*-mediated reporter enzyme activity is mainly localized in leaf tissue (**Figure 1B**). Of note, *NIMIN3* gene expression is independent from an intact *NPR1* gene (**Figure 2A**).

**Table 1:** Primers and control plasmids used in RT-PCR analyses.

Gene	Control plasmid	Primer	Sequence	Fragment size (bp)
*NIMIN1*	pGBT9/NIMIN1	N1fwd N1bck	5′-CGGGATCCATATGTATCCTAAACAATTTAG 5′-AACCCGGGCTACTACAATGCAAGATTAAGATC	449
*NIMIN2*	pGBT9/NIMIN2	N2fwd N2bck	5′-ACGCGTAGAAGAAGATAACGG 5′-CTAACGCTGTCTGGTTCCGGT	330
*NIMIN3*	pGBT9/NIMIN3	N3fwd N3bck	5′-GGGGATCCATATGGACAGAGACAGAAAGAG 5′-TTCCCGGGCTACAGAGAAAGATTCAAGTC	357
*PR-1*	pUC19/AtPR-1	PR1fwd PR1bck	5′-GGGGATCCATATGAATTTTACTGGC 5′-CTGAGCTCTTAGTATGGCTTCTCG	504
*Actin1*	–	Act1 Act2	5′-CGATGAAGCTCAATCCAAACGA 5′-CAGAGTCGAGCACAATACCG	302

### SALICYLIC ACID-MEDIATED INDUCTION OF *NIMIN1* AND *NIMIN2* PROCEEDS THROUGH SEPARATE PATHWAYS

RNA analyses as depicted in **Figure 1A** had shown that *NIMIN1* was expressed only after induction, just as *PR-1*, while *NIMIN2* expression was occasionally observed prior to chemical treatment of plants. This finding was unexpected since the *NIMIN2* promoter exhibits clear chemical induction in transgenic tobacco plants (0 GUS units and 265.0 GUS units on an average for water and SA treatment, respectively, *n* = 10; **Figure 1B**;Glocova et al., 2005). It therefore seemed of interest to analyze regulation of the *NIMIN1* and *NIMIN2* genes in closer detail.

Initially, we used two *npr1* mutants, *npr1-1* and *npr1-2*, which are not able to support *PR-1* gene induction (Cao et al., 1994;Glazebrook et al., 1996). Surprisingly, *NIMIN1*, like *PR-1*, was inactive in absence of a functional *NPR1* gene (**Figure 2A**). Yet, *NIMIN2* expression was clearly detectable in both *npr1* mutants, although, in some experiments, *NIMIN2* transcript levels appeared to accumulate to lower overall levels in *npr1* than in wild-type plants (**Figure 2A** and data not shown). Our data are in conflict with another report.Blanco et al. (2009) have described that expression of both *NIMIN1* and *NIMIN2* is abolished in the *npr1-1* mutant. To support our results, we verified the identity of the *NIMIN2* RT-PCR products by digestion with restriction enzymes (data not shown). Hence, *NIMIN2* expression, unlike *NIMIN1* and *PR-1* expression, may be either independent or only partly dependent on *NPR1*. Furthermore, the kinetics of gene induction turned out to be different between *NIMIN1* and *NIMIN2*. Both genes are expressed transiently after SA application (**Figure 2B**). Yet, *NIMIN2* gene expression started immediately (0.5 h) after SA treatment, reached its maximum early (after 1 h) and was maintained at a high level for 24 h (**Figure 2B**). Thus, *NIMIN2* seems to be an immediate early SA responsive gene, as suggested previously for the tobacco *NIMIN2a* gene (Horvath et al., 1998). *NIMIN1* transcripts, on the other side, became most abundant only around 2 h after SA application (**Figure 2B**). This is clearly later than the onset of *NIMIN2* expression, yet earlier than the onset of *PR-1* induction. Notably, *NIMIN1* expression appeared even more transient than *NIMIN2* expression and was already shut down when *PR-1* transcripts began to accumulate. The time course of *NIMIN1* gene induction shown here is in accordance with previous results obtained by northern blotting (Weigel et al., 2005). Together, our data strongly suggest that SA-mediated induction of the *NIMIN1* and *NIMIN2* genes proceeds through separate pathways.

The kinetics of gene induction were also monitored in tobacco seedlings containing *NIMIN_Pro_*::GUS reporter gene constructs. Transgenic seeds were germinated on SA-containing medium. The germination of seeds occurred simultaneously for all lines analyzed, and the development of seedlings progressed similarly. GUS enzyme activities were first determined 7 days after sowing when small seedlings had emerged. With both *NIMIN2_Pro_*::GUS and *NIMIN1_Pro_*::GUS, we did not observe a clear induction profile (**Figure 2C**). GUS enzyme activity was already switched on to high levels early after germination. In contrast, *PR-1a* promoter activation and accumulation of the endogenous PR-1 proteins occurred with significant delay (**Figure 2C**). Thus, the kinetics of reporter gene activation from the *NIMIN1* and *NIMIN2* promoters in SA-treated tobacco seem to parallel the transcript accumulation patterns observed in *Arabidopsis*, i.e., *NIMIN* genes are induced by SA prior to *PR-1* genes. The data indicate that the molecular cues for early induction during the SAR response are contained within the 1 kb 5′-flanking regions of *NIMIN1* and *NIMIN2*, and that these cues are recognized in the heterologous species tobacco. Reporter gene expression from both the *NIMIN1* and *NIMIN2* promoters occurred in leaf and root tissue (**Figure 1B**). Likewise, green fluorescent protein (GFP) expression from the 0.8 kb *NIMIN1* promoter has been observed in roots, petioles, and leaves in transgenic *Arabidopsis* plants (Fonseca et al., 2010). This expression pattern distinguishes the SA-inducible *NIMIN1* and *NIMIN2* promoters from the *NIMIN3* promoter and the tobacco *PR-1a* promoter which are predominantly active in leaf tissue (**Figure 1B**).

### NIMIN1 AND NIMIN3 SUPPRESS SALICYLIC ACID-INDUCED EXPRESSION FROM THE TOBACCO *PR-1a* PROMOTER

**FIGURE 3:**
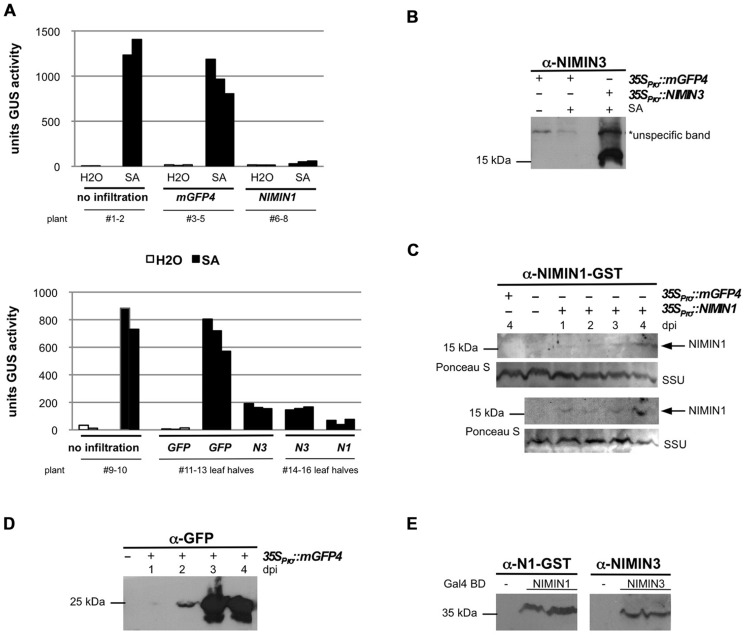
***Arabidopsis* NIMIN1 and NIMIN3 suppress salicylic acid-induced gene expression from the tobacco *PR-1a* promoter in *N.* benthamiana**. **(A)** Effects of transient expression of *35S_Pro_*::NIMIN1 and *35S_Pro_*::NIMIN3 in an *N. benthamiana* reporter line with integrated *-1533PR-1a_Pro_*::GUS. Three plants were infiltrated in parallel for each gene construct with *Agrobacterium* strains as indicated. For a better direct comparison, the two halves of the same leaf were infiltrated with Agrobacteria harboring *35S_Pro_*::NIMIN1 and *35S_Pro_*::NIMIN3, respectively. Leaf disks excised from infiltrated leaf areas were floated on water or on 1 mM SA before determination of GUS enzyme activity. The three bars for each construct and treatment represent GUS activities from the three agroinfiltration experiments performed in parallel. Representative results are shown. N1, NIMIN1; N3, NIMIN3. **(B)** Immunodetection of NIMIN3 in extracts from agroinfiltrated and SA-floated leaf tissue. NIMIN3 accumulation was detected with a specific antiserum in an extract shown in **Figure 3A**. An unspecific band marked on the X-ray serves as loading control. Exposure of the X-ray film was for 1 min. **(C)** Immunodetection of NIMIN1 after agroinfiltration. Results from two independent time course experiments are shown. Leaf tissue was extracted after infiltration as indicated. Extracts were analyzed for protein accumulation with a specific antibody. As loading control, the region of the nitrocellulose filters with the small subunit of RuBisCO (SSU) stained with Ponceau S is shown. Exposure of the X-ray films was over night. dpi, days post-infiltration. **(D)** Immunodetection of green fluorescent protein (GFP) after agroinfiltration. Leaf tissue was extracted after infiltration as indicated. Exposure of the X-ray film was for 1 min. **(E)** Immunodetection of NIMIN1- and NIMIN3-Gal4 DNA binding domain (GBD) fusion proteins in extracts from transformed yeast. The NIMIN1 and NIMIN3 fusions were detected with the specific antisera used in **Figures 3B,C**.

**FIGURE 4:**
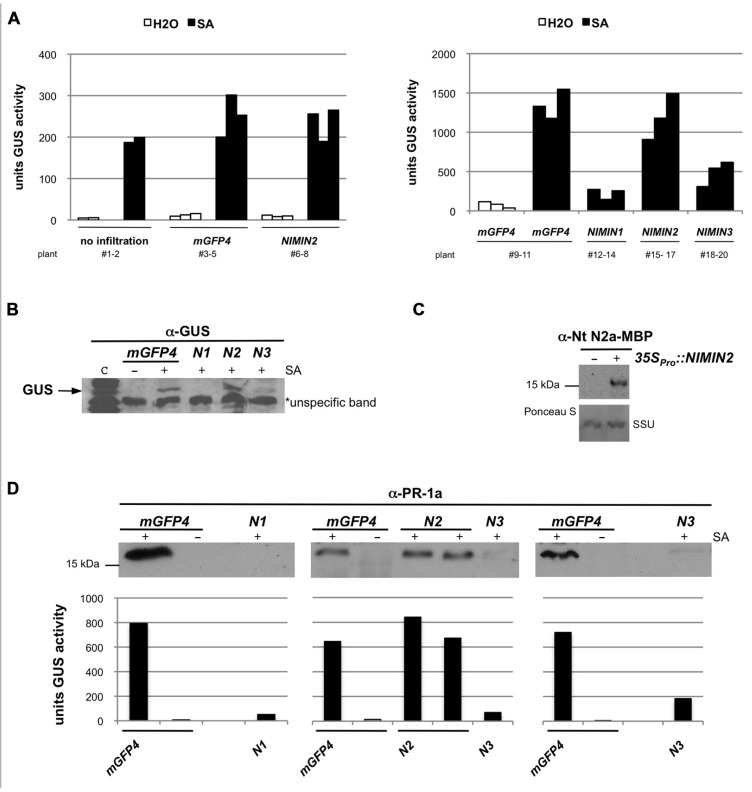
***Arabidopsis* NIMIN2 does not affect salicylic acid-induced gene expression from the tobacco *PR-1a* promoter in *N. benthamiana***. Transient expression assays and immunodetection were performed as described in **Figure 3**. N1, NIMIN1; N2, NIMIN2; N3, NIMIN3. **(A)** Effects of transient expression of *35S_Pro_*::NIMIN2 in the *N. benthamiana* *-*1533PR-1a_Pro_::GUS reporter line. The effects of NIMIN2 on the *PR-1a::GUS* reporter are compared to effects produced by NIMIN1 and NIMIN3. Representative results are shown. **(B)** Effects of transient expression of *35S_Pro_*::NIMIN1, *35S_Pro_*::NIMIN2, and *35S_Pro_*::NIMIN3 on accumulation of the GUS reporter protein in SA-treated leaf tissue. GUS accumulation was detected in extracts shown in **Figure 4A**. Lane c contains an extract from a tobacco plant stably transformed with *35S_Pro_*::GUS. An unspecific band marked on the X-ray serves as loading control. **(C)** Immunodetection of NIMIN2 in agroinfiltrated tissue. NIMIN2 accumulation was detected with a specific antiserum in an extract shown in **Figure 4A**. **(D)** Effects of transient expression of *35S_Pro_*::NIMIN1, *35S_Pro_*::NIMIN2, and *35S_Pro_*::NIMIN3 on accumulation of the endogenous PR-1 protein in SA-treated *N. benthamiana* leaf tissue. GUS reporter enzyme activities of extracts analyzed for PR-1 protein accumulation are given below the immunodetections.

To unravel the functional significance of *NIMIN* gene expression at different times during the SAR response, we have developed an *in planta* assay for NIMIN activity. The gene coding for *GUS* under control of the tobacco *PR-1a* promoter (*-1533PR-1a_Pro_*::GUS;Grüner and Pfitzner, 1994) was stably integrated in the genome of *Nicotiana benthamiana*. Several primary transformants were obtained all of which exhibited very strong and stringent induction of the reporter gene upon SA treatment of leaf tissue (data not shown). One typical line (3 GUS units uninduced and 1100 GUS units after SA treatment) was propagated, and T2 plants were used for infiltration experiments with an *Agrobacterium* strain carrying the gene for GFP (*mGFP4*) driven by the *Cauliflower mosaic virus* (CaMV) *35S RNA* promoter (*35S_Pro_*::mGFP4). Infiltration of *35S_Pro_*::mGFP4 Agrobacteria yielded GUS enzyme activities only slightly above the background levels of non-infiltrated control leaves, showing that agroinfiltration alone is not sufficient for efficient activation of the *PR-1a_Pro_*::GUS reporter gene (**Figures 3A** and **4A**).

Next, we tested the influence of different NIMIN proteins on *PR-1a* gene induction after agroinfiltration of *N. benthamiana*. It has been shown previously that overexpression of *NIMIN1* suppresses SA-mediated *PR* gene induction and SAR in transgenic *Arabidopsis* plants (Weigel et al., 2005). However, the functional roles of NIMIN2 and NIMIN3 are not known. Initially, Agrobacteria adjusted to equal cell densities were infiltrated into leaves of individual *N. benthamiana* plants with the *-1533PR-1a_Pro_*::GUS reporter. In each experiment, three plants were infiltrated in parallel with the same *Agrobacterium* strain. After 4–5 days, disks were cut from leaf areas close to the infiltration sites. At this time, strong fluorescence was typically observed in tissue infiltrated with *35S_Pro_*::mGFP4 Agrobacteria, demonstrating efficient expression of the *GFP* reporter. GUS activity assays revealed that none of the NIMIN proteins is able to activate the *PR-1a_Pro_*::GUS reporter gene on its own (**Figures 3A** and **4A** and data not shown). The excised leaf disks were then floated for 2 days on water or on a 1 mM SA solution. As controls, disks from non-agroinfiltrated leaves were incubated on water and SA. After floating, proteins were extracted from leaf tissue, and GUS reporter activity was determined. In other experiments, we have infiltrated the two halves of a single leaf with *Agrobacterium* strains harboring different constructs in order to allow an even more direct comparison between effects exerted by the respective NIMIN proteins. Consistent with what has been described for *NIMIN1* overexpression in transgenic *Arabidopsis* plants, agroinfiltration of *35S_Pro_*::NIMIN1 bacteria suppressed SA-mediated *PR-1a* promoter activation to nearly background levels as compared to GUS levels observed in *GFP* expressing leaf disks floated on water (**Figures 3A** and **4A**). Quite surprisingly, *NIMIN3* overexpression, too, clearly repressed *GUS* reporter gene induction from the Nt *PR-1a* promoter in *N. benthamiana* (**Figures 3A** and **4A**). Repression with NIMIN3 was, however, weaker than with NIMIN1 (**Figures 3A** and **4A**). The presence of NIMIN1 and NIMIN3 proteins in infiltrated *N. benthamiana* leaf tissue was monitored by immunodetection using specific antisera. NIMIN3 accumulated to high levels. The protein was readily detected in extracts from SA-floated leaf disks and also in extracts from agroinfiltrated tissue without SA induction (**Figure 3B** and data not shown). In contrast, we were not able to detect *NIMIN1* expression in extracts from SA-treated leaf tissue. We therefore performed time course experiments monitoring NIMIN1 accumulation in twofold concentrated extracts from 1 to 4 days after agroinfiltration. Whereas GFP accumulated to high levels at 3 and 4 days post-inoculation (dpi; **Figure 3D**), NIMIN1 protein was detected only faintly (**Figure 3C**). The inability to detect high amounts of NIMIN1 in agroinfiltrated plant tissue is, however, not due to a low sensitivity of the anti-NIMIN1 serum we used. Detection of NIMIN1 and NIMIN3-Gal4 DNA binding domain (GBD) fusion proteins, which are expressed to similar levels in yeast (Weigel et al., 2001), was similar for both NIMIN3 and NIMIN1 with the specific antisera (**Figure 3E**).

### NIMIN2 DOES NOT SIGNIFICANTLY AFFECT SALICYLIC ACID-INDUCED EXPRESSION OF TOBACCO *PR-1* GENES

Likewise surprisingly, agroinfiltration of the *N. benthamiana* reporter line with *35S_Pro_*::NIMIN2 harboring bacteria did not repress SA-mediated induction of the *PR-1a_Pro_*::GUS transgene (**Figures 4A,B**). Expression of *NIMIN2* in *N. benthamiana* leaf tissue was demonstrated by immunodetection using a specific antiserum directed against Nt NIMIN2a-maltose binding protein (MBP) which exhibits cross-reactivity with *Arabidopsis* NIMIN2 (**Figure 4C**). Thus, albeit similar to each other and possessing similar NPR1 interaction motifs, NIMIN2 and NIMIN1 seem to fulfill different, even opposing, functions in the SA signal transduction pathway.

We also tested whether transient expression of At *NIMIN* genes in *N. benthamiana* is able to suppress induction of endogenous *PR-1* genes. *N. benthamiana* (Nb) carries a gene for a basic PR-1 protein. The amino acid sequence for the basic PR-1 protein is co-linear with *N. tabacum* acidic PR-1 proteins except for a 19 amino acid-long extension at the C-terminus of Nb PR-1. In the co-linear region, the identity (similarity) between the basic Nb PR-1 protein and Nt PR-1a is 64% (87%). Consequently, using an antiserum raised against Nt PR-1a, we were able to detect a PR-1-related protein exhibiting a slightly higher molecular weight than the acidic Nt PR-1 proteins in extracts from *N. benthamiana* leaf disks floated on 1 mM SA (data not shown). SA induction of this protein was clearly suppressed in *N. benthamiana* tissue overexpressing *NIMIN1* or *NIMIN3*, but not in tissue overexpressing *NIMIN2* (**Figure 4D**).

### *Arabidopsis* NIMIN PROTEINS CANNOT BIND SIMULTANEOUSLY TO NPR1 IN YEAST

**FIGURE 5:**
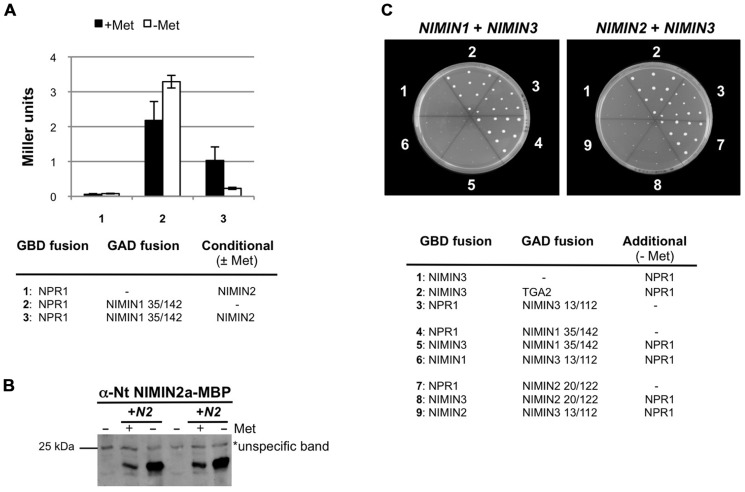
***Arabidopsis* NIMIN1, NIMIN2, and NIMIN3 do not bind simultaneously to At NPR1 in yeast**. **(A)** Yeast two-hybrid interaction of At NPR1-Gal4 DNA binding domain (GBD) and NIMIN1-Gal4 activation domain (GAD) fusion proteins in absence and presence of NIMIN2. *NIMIN2* was expressed from the *Met25* promoter which is repressed in presence and de-repressed in absence of methionine. **(B)** Immunodetection of NIMIN2 in yeast. Yeast cells analyzed for *lacZ* reporter gene expression in **Figure 5A** were probed for accumulation of NIMIN2 protein. N2, NIMIN2. **(C)** Yeast three-hybrid interaction of At NPR1 with NIMIN1 and NIMIN3 or with NIMIN2 and NIMIN3. NIMIN1, NIMIN2, and NIMIN3 were expressed as fusions with the GBD or GAD. Simultaneous interaction of At NPR1 with GAD-TGA2 and GBD-NIMIN3 serves as positive control for formation of a ternary protein complex.

**FIGURE 6:**
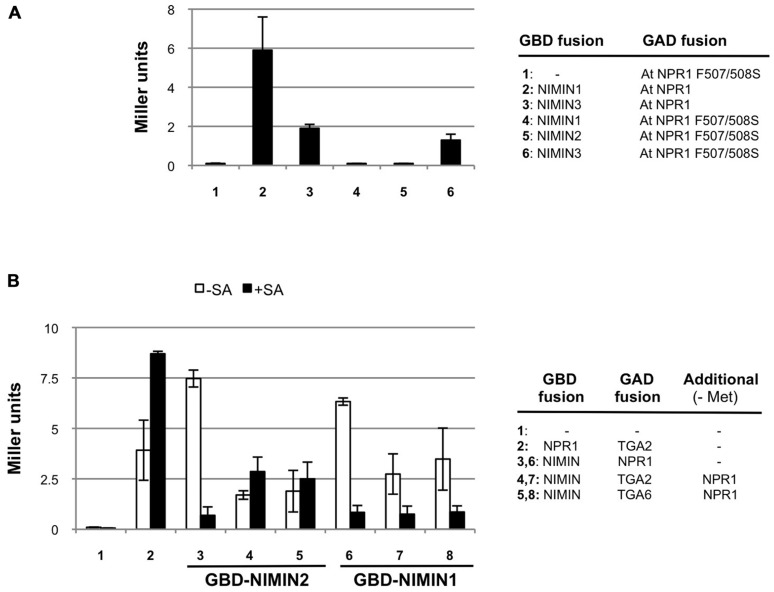
***Arabidopsis* NIMIN1 and NIMIN2 interact differentially with At NPR1 in yeast**. **(A)** Yeast two-hybrid interaction of NIMIN1, NIMIN2 or NIMIN3 expressed as GBD fusions with the mutant protein At NPR1 F507/508S expressed as GAD fusion. Interactions of GAD-At NPR1 with GBD-NIMIN1 and GBD-NIMIN3 serve as positive controls. **(B)** Effect of SA on formation of ternary protein complexes comprising GBD-NIMIN1 or GBD-NIMIN2, GAD-At TGA2 or GAD-At TGA6 and At NPR1. The binary interactions of At NPR1 with NIMIN1 or NIMIN2 or TGA2 serve as controls for effects of SA (concentration 0.3 mM) on the NPR1–NIMIN1/2 interaction.

Differential regulation of *NIMIN* genes and differential effects of NIMIN proteins on *PR-1* induction strongly suggested that NIMINs serve unique functions at specific time points during the SAR response in *Arabidopsis*, an assumption fully consistent with our previous observation that NIMIN3 and NIMIN1/NIMIN2 bind to physically separate regions of At NPR1 (Weigel et al., 2001). Therefore, it was of interest to test whether NIMIN proteins are able to bind simultaneously to NPR1, or whether their binding excludes each other. To address this question, we made use of a yeast three-hybrid (Y3H) system. In this assay, interaction of two proteins can be monitored at different concentrations of a third protein whose expression level is controlled by methionine (Met) in the growth medium (Tirode et al., 1997). Previously, we have demonstrated that NIMIN proteins are able to interact with TGA transcription factors in presence of NPR1 (**Figures 5C** and **6B**;Weigel et al., 2001), showing that NIMINs and TGA factors possess independent binding sites on NPR1 which can be occupied at the same time. The same assay was used for monitoring binding of two different NIMIN proteins to NPR1. To this end, we used partial *NIMIN* cDNA clones which we had isolated in a yeast two-hybrid (Y2H) screen with the At NPR1 bait (Weigel et al., 2001).

Initially, we tested whether NIMIN1 and NIMIN2 can bind together to NPR1 in Y3H assays. Both proteins possess similar NPR1 interaction motifs by which they bind to the C-terminus of NPR1 (Weigel et al., 2001; **Figure 6A**). Truncated NIMIN1 or NIMIN2 including their NPR1 interaction motif were expressed as fusions with the Gal4 transcription activation domain (GAD), and full-length *NIMIN1* or *NIMIN2* were expressed from the *Met25* promoter, which is repressed in presence and de-repressed in absence of methionine. NPR1 was expressed as GBD fusion. The interactions of NIMIN1 or NIMIN2 with NPR1 were disrupted in presence of NIMIN2 or NIMIN1, respectively (**Figure 5A** and data not shown). Furthermore, complex formation between NPR1 and NIMIN1 was clearly dependent on the concentration of NIMIN2 (**Figures 5A,B**). Together, the data suggest that NIMIN1 and NIMIN2 may compete for the same binding site on NPR1.

Next, we asked whether NIMIN1 or NIMIN2 can bind to NPR1 in presence of NIMIN3 which interacts with NPR1 via a site distant from the NIMIN1/NIMIN2 binding site (Weigel et al., 2001). NIMIN1 and NIMIN3 or NIMIN2 and NIMIN3 were expressed as GBD or GAD fusions, while *NPR1* was expressed from the de-repressed *Met25* promoter. Surprisingly, the interaction between NIMIN1 and NPR1 and between NIMIN2 and NPR1 was disrupted in presence of NIMIN3 (**Figure 5C**). Hence, NIMIN3 binding to NPR1 seems to inhibit NIMIN1/NIMIN2 interaction, and simultaneous binding of NIMIN1, NIMIN2, and NIMIN3 to NPR1 may exclude each other.

### NIMIN1 AND NIMIN2 INTERACT DIFFERENTIALLY WITH NPR1

In tobacco NPR1, binding of NIMIN2 proteins occurs in the region from amino acids 494 to 510 (Maier et al., 2011). The domain is highly conserved in NPR1 proteins from many plant species, including *Arabidopsis*, regarding both the sequence and its position within the amino acid chain (for At NPR1 94% identity, 100% similarity, from amino acids 496 to 512). To test whether both NIMIN1 and NIMIN2 bind to this region in At NPR1 and whether binding occurs in a similar fashion, we introduced mutations F507S and F508S into At NPR1. Nt NPR1 F505/506S is no longer able to interact with Nt NIMIN2a or Nt NIMIN2c (Maier et al., 2011). Similarly, mutation of F507/508S completely abolishes binding of NIMIN1 and NIMIN2 to At NPR1, but not binding of NIMIN3 (**Figure 6A**).

We then analyzed the relations of NPR1 with NIMIN1 and NIMIN2 in ternary protein complexes including TGA transcription factors. We have shown previously that SA administered to growth medium impairs formation of NPR1–NIMIN1 and NPR1–NIMIN2 complexes in Y2H assays, and that the sensitivity of loss of protein–protein interaction is very similar for both NIMIN1 and NIMIN2 (IC_50_ ≈ 20 μM SA;Maier et al., 2011). Here, we monitored effects of SA on NPR1–NIMIN1 and NPR1–NIMIN2 interactions in presence of TGA2 or TGA6. Interaction of *Arabidopsis* NPR1 with TGA factors is not diminished with SA (**Figure 6B**;Maier et al., 2011). Ternary complexes comprising NIMIN1 were sensitive to SA as observed before for the NIMIN1–NPR1 binary interaction (**Figure 6B**). Quite surprisingly, however, ternary complexes comprising NIMIN2 proved to be stable in presence of SA (**Figure 6B**). Thus, although possessing similar NPR1 interaction motifs and binding to the same site in the C-terminus of NPR1, NIMIN1, and NIMIN2 can form complexes with NPR1 and TGA factors exhibiting differential sensitivity to SA, implying that these two NIMIN proteins interact differentially with NPR1 in transcription complexes on *PR* gene promoters.

## DISCUSSION

NIM1-INTERACTING proteins have been identified through a Y2H screen with *Arabidopsis* NPR1 as bait. Although of rather small molecular weight, the proteins share several conserved regions with each other which are likely of functional relevance. Thus, all NIMIN proteins encompass an LxLxL/EAR (ethylene-responsive element binding factor-associated amphiphilic repression) motif at their C-terminus, and NIMIN1 and NIMIN2 possess a common motif for interaction with a domain in the C-terminus of *Arabidopsis* and tobacco NPR1. On the other hand, NIMIN1 and NIMIN3 have been reported to share a conserved PA/SFQPEDF signature (Weigel et al., 2001), suggesting that NIMIN1 and NIMIN3, albeit binding to different regions of NPR1, may exert similar activities. To understand the action of related, yet distinct, NIMIN proteins on NPR1, we have performed a comparative analysis of *Arabidopsis* NIMIN1, NIMIN2, and NIMIN3. We have studied the expression profiles of *NIMIN* genes, the effects of NIMIN proteins on SA induction of the SAR marker *PR-1* and their interaction with NPR1. Our results suggest that the *Arabidopsis* NIMIN proteins exert unique and complementary functions on NPR1 at different stages of the SAR response.

### NIMIN3 REPRESSES *PR-1* IN UNCHALLENGED PLANTS

As opposed to *NIMIN1* and *NIMIN2*, which are clearly responsive to SA, *NIMIN3* is expressed constitutively at a low level in *Arabidopsis* leaf tissue. In our current work, we have not found any indications for enhancement of *NIMIN3* expression by SA or other plant defense hormones. Most importantly, the *NIMIN3* promoter is weakly active in leaf tissue and does not respond to the SAR signal molecule SA. Hence, NIMIN3 is likely to function on a constitutive basis in unchallenged plants before the induction of SAR. This idea is consistent with our previous finding that NIMIN3 does not possess the interaction site by which NIMIN1 and NIMIN2 bind to the SA-sensitive NPR1 C-terminus (Weigel et al., 2001;Maier et al., 2011). When transiently overexpressed in the *N. benthamiana -1533PR-1a::GUS* reporter line created by us, NIMIN3, like NIMIN1, is able to suppress SA-induced activation of the reporter. Similarly, NIMIN3, like NIMIN1, also suppresses induced expression of an endogenous *PR-1* gene in *N. benthamiana*. Altogether, repression effects exerted by NIMIN3 in *N. benthamiana* seem moderate, when compared to effects observed with NIMIN1. On the other side, we did not expect suppression of *PR-1* gene induction to occur at all by NIMIN3 in *Nicotiana* species. First, a true NIMIN3 homolog has not been identified to date from tobacco or tomato. Furthermore, NPR1 family members from tobacco, Nt NPR1 and Nt NPR3, have not been found to interact with NIMIN3 in Y2H assays (Zwicker et al., 2007;Maier et al., 2011), whereas NIMIN3 clearly interacts with *Arabidopsis* NPR1 (Weigel et al., 2001). Thus, the biochemical basis of NIMIN3-mediated suppression of *PR-1* in *N. benthamiana* is not clear. However, we have noted previously that NIMIN3 and NIMIN1 share the conserved amino acid signature PA/SFQPEDF (from here on termed EDF motif;Weigel et al., 2001). This signature is also present in the rice (Os) NIMIN homolog NRR and some of its paralogs (consensus sequence WRP-F-W/MEDF;Chern et al., 2012). Mutations of NRR and its paralogs in this region have uncovered the motif as domain for strong interaction with rice NH1/NPR1 causing repression of transcription activity of Os NH1/NPR1 in a rice transient assay system. In contrast, the motif mediates only very weak interaction between NRR and *Arabidopsis* NPR1 (Chern et al., 2012). We have introduced mutations in the EDF motifs of NIMIN3 and NIMIN1 (E63A D64V in NIMIN3; E94A D95V in NIMIN1), and tested activities of the mutant proteins in Y2H assays with Gal4 AD-At NPR1 and in the *N. benthamiana* transient assay system. Unfortunately, the mutant proteins did not accumulate to detectable levels, neither in yeast nor in plant tissue, and therefore, the significance of the EDF domain for NIMIN3 and NIMIN1 could not be assessed (Masroor and Pfitzner, unpublished data). It is of interest, however, to note that binding of At NPR1 to NIMIN3 occurs within the 60 amino acid-long C-terminal half including the EDF motif (Weigel et al., 2001). Given the conservation of the amino acid sequence in NPR1 interactors from multiple plant species and the clear results in the rice system reported byChern et al. (2012), we infer that the EDF signature is functional in *Arabidopsis* NIMINs, and that the domain is involved in regulation of *PR* genes via the NIMIN–NPR1 complex. The significance of the EDF domain for *PR* gene induction may, however, vary among different plant species. In this line, suppression of *PR-1* induction in *N. benthamiana* may be mediated via the EDF domain in NIMIN3 and NIMIN1, and suppression by NIMIN1 would be stronger because NIMIN1, unlike NIMIN3, can interact via a second domain with the NPR1 C-terminus. Of note, several cDNAs from *N. tabacum* and *N. benthamiana* coding for NIMIN proteins with the EDF motif (consensus WNL/PA/TF/L-T/PEDF) have been described in the databanks, underscoring our assumption that the EDF domain may have functional relevance also in tobacco. The mechanism by which the EDF domain in NIMIN proteins could suppress *PR-1* gene induction remains, however, elusive. Alternatively, suppression of *PR-1* induction in *N. benthamiana* by NIMIN3 and NIMIN1 may occur via the C-terminal LxLxL/EAR motif which has been implicated in recruiting the transcriptional co-repressor TOPLESS (*Arabidopsis* Interactome Mapping Consortium, 2011). In summary, our data would support the view that NIMIN3 can target the NPR1 complex in tobacco, and that NIMIN3 is a repressor of inadvertent *PR-1* gene expression in unchallenged *Arabidopsis* leaf tissue.

### NIMIN2 DOES NOT AFFECT SALICYLIC ACID INDUCTION OF *PR-1*

We have noted previously that *NIMIN2* is responsive to SA (Weigel et al., 2001;Glocova et al., 2005). Here, using RT-PCR analyses, we show that *NIMIN2* mRNA accumulates very early after treatment of plants with SA, and, in several cases, *NIMIN2* mRNA was already detectable in plant tissue without exposure to chemicals at all. From our observations, we conclude that *NIMIN2* is more readily induced than *NIMIN1* or *PR-1*, consistent with the finding that *NIMIN2* expression, as opposed to *NIMIN1* and *PR-1* expression, is independent from an intact *NPR1* gene requiring activation by SA. Surprisingly, overexpression of *NIMIN2* in the *N. benthamiana -1533PR-1a::GUS* reporter line does not appear to have an effect on SA-induced *PR-1* gene expression. This finding is consistent with our previous observation showing that overexpression of a *NIMIN2 *homolog, Nt *NIMIN2a*, in transgenic tobacco plants did not result in massive *PR-1* repression as reported in similar experiments for At *NIMIN1* and Os *NRR* overexpression (Chern et al., 2005, 2008;Weigel et al., 2005). Hence, NIMIN2 is likely to play a role at the very onset of SAR and is unlikely to be involved in repression of *PR-1* gene induction.

### NIMIN1 CONTROLS EXPRESSION OF LATE SAR-INDUCED *PR-1*

*NIMIN1* is an early SA-activated and *NPR1*-dependent gene which is induced after *NIMIN2*, but clearly before *PR-1*. *NIMIN1* is expressed only transiently, and the NIMIN1 protein does not appear to accumulate to high levels. These features are compatible with a role of NIMIN1 as regulator of late SAR genes, e.g., *PR-1*, preventing their premature activation. The repression effect exerted by NIMIN1 in the *N. benthamiana -1533PR-1a::GUS* reporter line is very strong. Above, we have argued that *PR-1* repression may be mediated via the EDF domain in NIMIN1, although we were not able to provide direct proof for this assumption. It is important to note, however, that NIMIN2 does not possess the EDF motif and does not repress *PR-1* gene induction in our system. Curiously, although not accumulating to substantial levels in agroinfiltrated *N. benthamiana* leaf tissue, NIMIN1 executes strong effects raising the question how NIMIN1 could suppress *PR-1* gene expression in near physical absence? Different scenarios seem conceivable. For example, NIMIN1 could be stable and exert its function only in direct association with NPR1. Any excess NIMIN1 protein would immediately be degraded. In this scenario, NIMIN1 could act to prohibit contact of NPR1 to downstream transcription factors either by sterical hindrance or, in imitation to the action of a chaperone, by imposing a non-productive bent on NPR1. Together, our data support a view where NIMIN1 acts only later during the SAR response, after NIMIN2, keeping tight control over *PR-1* by promoting its repression. Notably, we were not able to detect simultaneous binding of NIMIN3, NIMIN2, or NIMIN1 to NPR1, and we found that NIMIN1 and NIMIN2 bind differentially to NPR1 in ternary protein complexes including TGA transcription factors.

### WORKING MODEL FOR THE CONSECUTIVE ACTION OF *Arabidopsis* NIMIN PROTEINS IN THE COURSE OF SAR

**FIGURE 7:**
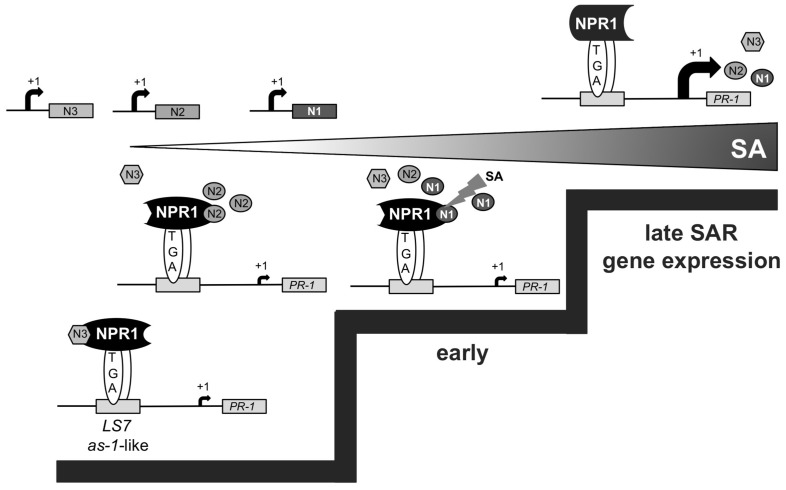
**Working model for the consecutive action of *Arabidopsis* NIMIN proteins in the course of SAR**. The model implies sequential interaction between diverse NIMIN proteins and NPR1 to form regulatory complexes with differential biochemical capacities in the course of SAR. The model also suggests that sensing of ambient SA levels in diseased plants may occur through the various NIMIN–NPR1 complexes, enabling activation of *PR* genes at distinct threshold levels of SA (indicated by steps). In this scenario, the defense gene *PR-1* is induced late during SAR by direct action of SA on the NIMIN1–NPR1 regulatory complex.

Based on our findings, we propose sequential formation of different NIMIN–NPR1 complexes to promote defense gene activation at distinct stages of SAR (**Figure 7**). While NIMIN3 represses inadvertent *PR* gene activation in unchallenged plants, NIMIN2 is induced at low tissue levels of SA to relieve NIMIN3 repression by binding to the NPR1 C-terminus. This process may allow activation of early SA- and *NPR1*-dependent genes, e.g., *NIMIN1*. Interaction of NIMIN2 with the NPR1 C-terminus does not, however, appear to be sufficient to activate substantial expression of the late SAR gene *PR-1*. NIMIN2 action on NPR1 is transient and is followed by NIMIN1 replacing NIMIN2. NIMIN1 suppresses activation of *NPR1*-dependent SAR genes. NIMIN1 action on NPR1 seems even more transient than NIMIN2 action, and instability of NIMIN1 protein would be a crucial prerequisite for relief of *PR-1* gene repression. In this scenario, late SAR genes would be activated through direct action of SA on NPR1 (Maier et al., 2011;Fu et al., 2012;Wu et al., 2012) causing removal of repressing NIMIN1 from the NPR1 complex (Maier et al., 2011). In conclusion, consecutive action of NIMIN proteins with different biochemical capacities on the central SAR regulator NPR1 is needed to ensure sudden, strong and coordinate expression of defense genes to successfully combat invading pathogens. In this line, the NIMIN–NPR1 connection may constitute a molecular device to monitor ambient SA levels in diseased plants, enabling the plant to translate a steadily increasing gradient of the defense hormone SA into two clear decision steps, early and late SAR gene expression.

## MATERIALS AND METHODS

### DNA CONSTRUCTS

For transient gene expression assays, the coding regions from *NIMIN1*, *NIMIN2*, and *NIMIN3* were inserted as *Bam*HI/*Sac*I fragments into pBin19/35S_ Pro_::GUS (Jefferson et al., 1987) from which the *GUS* reporter gene had been excised. The coding regions were amplified from the respective pGBT9 plasmids (Weigel et al., 2001) using C-terminal primers with the native stop codons and a *Sac*I restriction endonuclease site added 3′ to the stop codons.

The *NIMIN3_Pro_*::GUS reporter gene was constructed in analogy to the *NIMIN1_Pro_*::GUS and *NIMIN2_Pro_*::GUS chimeric genes (Glocova et al., 2005). The *NIMIN3* promoter sequence was amplified from *Arabidopsis thaliana* (L.) Heynh. Col-0 genomic DNA using primers N3-P2 (5′-TTAAGCTTATACGGGACATAGTGCACAGCC) and N3-P1 (5′-AAGGATCCTGAACCGCTCTCTCTTCCTTCC). N3-P1 primes immediately upstream of the ATG translation start codon of *NIMIN3*. The resulting 1.4 kb fragment was ligated to *Hin*dIII/*Bam*HI cleaved pBin19/35S_Pro_::GUS from which the *35S RNA* promoter had been removed.

To map the NIMIN1/NIMIN2 binding site in At NPR1, Phe-507 and Phe-508 were mutated to Ser using overlap extension PCR (Ho et al., 1989). The primers for mutagenesis were AtNPR1-14 (5′-CTCGGGAAACGAAGCAGCCCGCGCTGTTC) and AtNPR1-15 (5′-GAACAGCGCGGGCTGCTTCGTTTCCCGAG). The mutations were inserted in a C-terminal fragment of At *NPR1*. To this clone, the N-terminal At *NPR1* sequence was added as a 1.4 kb *Bam*HI/*Dra*III fragment, and the complete mutant sequence was ligated to *Bam*HI/*Sal*I cleaved pGBT9 and pGAD424.

All clones generated by PCR amplification were verified by DNA sequence analysis.

### RNA ISOLATION AND RT-PCR ANALYSES

RNA isolation and RT-PCR analyses were performed as described byZwicker et al. (2007). The primer combinations and control plasmids used for the different gene fragments are listed in Table 1. For the time course experiment shown in **Figure 2B**, RT-PCR assays were conducted to give approximately equal amounts of reaction products in order to enable direct comparison of *NIMIN1*, *NIMIN2*, and *PR-1* transcript accumulation at different time points after treatment of *Arabidopsis* with SA. To this end, RNAs were diluted 1:20 for RT-PCR amplification of *NIMIN2* transcripts.

### GENERATION AND CULTIVATION OF TRANSGENIC PLANTS

Transformation of tobacco (*N. tabacum* L. cv. Samsun NN) by *Agrobacterium tumefaciens* was performed according toGrüner et al. (2003). Tobacco lines with *PR-1a_Pro_*::GUS, *35S_Pro_*::GUS, *NIMIN1_Pro_*::GUS, and *NIMIN2_Pro_*::GUS have been described earlier (Grüner et al., 2003;Glocova et al., 2005). For localization of GUS enzyme activity *in situ* (**Figure 1B**) and for determination of SA-induced GUS activity in time course experiments (**Figure 2C**), seeds from transgenic tobacco were sown on MS medium with 400 μg ml^-^^1^ kanamycin or on selective medium supplemented with 0.3 mM SA.

### *Agrobacterium*-MEDIATED TRANSIENT GENE EXPRESSION IN *NICOTIANA BENTHAMIANA*

The *-1533PR-1a_Pro_*::GUS gene construct (Grüner et al., 2003) was integrated via *Agrobacterium*-mediated transformation into the genome of *N. benthamiana* Domin. All primary transformants exhibited strong and stringent induction of the *GUS* reporter gene in response to SA. A line with an intermediate GUS enzyme activity was propagated by selfing, and plants of the T2 generation were used for agroinfiltration experiments.

The pBin19 gene constructs were transferred by triparental mating to *Agrobacterium tumefaciens* strain LBA4404. Recombinant *Agrobacterium* strains were grown at 30°C in minimal medium supplemented with 50 μg ml^-^^1^ kanamycin and 50 μg ml^-^^1^ rifampicin to stationary phase. Cells were collected by centrifugation and resuspended in 10 mM MgCl_2_ and 150 μM acetosyringone to give an optical density (OD_600_) of 0.5 for all strains. Agrobacteria were incubated for 2–3 h at room temperature before agroinfiltration. To suppress post-transcriptional gene silencing, the bacterial suspensions were mixed with an equal volume of a strain carrying the p19 suppressor from *Tomato bushy stunt virus* (Voinnet et al., 2003). Four to six week-old greenhouse-grown *N. benthamiana* plants with integrated *-1533PR-1a_Pro_*::GUS were agroinfiltrated in the abaxial air spaces. To allow for a direct comparison between effects produced by different *NIMIN* strains, leaves at the same position on the axis of different plants or the two halves of the same leaf were injected. In each experiment, three independent plants were infiltrated with the same *Agrobacterium* suspension, and plants infiltrated with a strain containing *35S_Pro_*::mGFP4 (Haseloff et al., 1997) were used to control gene expression levels in leaf tissue. Expression of *GFP* was monitored under UV light. GFP fluorescence remained always strictly confined to infiltrated leaf areas. Agroinfiltrated tissue was processed 4 or 5 days post-infiltration (dpi), when strong GFP fluorescence was observed. At this point of time, bacterial titers were similar in leaf tissue agroinfiltrated with strains *35S_Pro_*::mGFP4, *35S_Pro_*::NIMIN1, or *35S_Pro_*::NIMIN2 (Wöhrle and Pfitzner, unpublished data). Furthermore, co-overexpression of *mGFP4* and *NIMIN1* produced the same levels of GFP fluorescence and of GFP protein accumulation as overexpression of *mGFP4* alone (Masroor and Pfitzner, unpublished data).

### *GUS* REPORTER GENE ASSAYS AND IMMUNODETECTION OF PROTEIN ACCUMULATION

Determination of GUS enzyme activity and histochemical localization of GUS activity *in situ* were performed as described previously (Weigel et al., 2001;Glocova et al., 2005). GUS activity is given in units (1 unit = 1 nmol 4-MU per hour per mg protein). For the time course experiment shown in **Figure 2C**, GUS enzyme activities were determined from pools of 10 seedlings for each data point. The same extracts were used for immunodetection of endogenous PR-1 proteins. Equal amounts of protein were loaded in each lane of the sodium dodecyl sulfate (SDS) gels.

To determine GUS enzyme activity after transient expression of *NIMIN* genes in *N. benthamiana*, two leaf disks each were punched out from non-infiltrated control or from agroinfiltrated leaf tissue at 4 or 5 dpi. Disks were floated for 2 days on water or on 1 mM SA and thereafter extracted with 150 μl GUS lysis buffer. The SA-induced reporter gene expression from the *PR-1a* promoter was compared in non-agroinfiltrated leaf tissue and in tissue infiltrated with *35S_Pro_*::mGFP4 and *35S_Pro_*::NIMIN chimeric genes. The same extracts were used for immunodetection of protein accumulation.

Immunodetection of proteins separated by SDS gel electrophoresis was performed as described earlier (Zwicker et al., 2007). Specific antisera were raised in rabbits immunized with *E. coli* expressed and purified proteins NIMIN1-GST, Nt NIMIN2a-MBP, and NIMIN3 according to standard procedures. PR-1 protein accumulation in *N. benthamiana* was detected with a specific antiserum against Nt PR-1a. For detection of GFP and GUS proteins, rabbit polyclonal antisera were used as recommended by the manufacturers (Santa Cruz Biotechnology and Abcam, respectively). To analyze accumulation of NIMIN1 at different times after agroinfiltration (**Figure 3C**), four leaf disks were harvested directly from each infiltrated tissue and extracted with 150 μl GUS lysis buffer yielding twofold concentrated extracts. SA induction of the GUS reporter protein and of an endogenous *N. benthamiana* PR-1 protein was compared in tissue infiltrated with *35S_Pro_*::mGFP4 and *35S_Pro_*::NIMIN chimeric genes. Equal extract volumes were loaded in each lane of an SDS gel. The loading of SDS gels for immunodetection of protein accumulation was checked by staining the nitrocellulose filters with Ponceau S (0.1% in 5% acetic acid). Alternatively, unspecific bands reacting with the antisera used are marked for demonstration of equal gel loading.

### YEAST TWO-HYBRID AND THREE-HYBRID ASSAYS

Yeast two-hybrid and yeast three-hybrid analyses in absence and presence of SA were conducted as reported earlier (Weigel et al., 2001;Maier et al., 2011). *LacZ* reporter gene activities are given in Miller units. Most plasmids used in the protein–protein interaction assays have been described (Weigel et al., 2001). pGAD10/NIMIN1 35/142, pGAD10/NIMIN2 20/122, and pGAD10/NIMIN3 13/112 encode NIMIN proteins truncated at their N-terminus. The plasmids were isolated in a Y2H screen with the At NPR1 bait (Weigel et al., 2001).

## Conflict of Interest Statement

The authors declare that the research was conducted in the absence of any commercial or financial relationships that could be construed as a potential conflict of interest.
